# Detection of fraud in lime juice using pattern recognition techniques and FT‐IR spectroscopy

**DOI:** 10.1002/fsn3.2260

**Published:** 2021-03-24

**Authors:** Amirhossein Mohammadian, Mohsen Barzegar, Ahmad Mani‐Varnosfaderani

**Affiliations:** ^1^ Department of Food Science and Technology Tarbiat Modares University Tehran Iran; ^2^ Department of Analytical Chemistry Tarbiat Modares University Tehran Iran

**Keywords:** artificial neural networks, classification, FT‐IR spectroscopy, lime juice, modeling

## Abstract

The lime juice is one of the products that has always fallen victim to fraud by manufacturers for reducing the cost of products. The aim of this research was to determine fraud in distributed lime juice products from different factories in Iran. In this study, 101 samples were collected from markets and also prepared manually and finally derived into 5 classes as follows: two natural classes (*Citrus limetta*, *Citrus aurantifolia*), including 17 samples, and three reconstructed classes, including 84 samples (made from Spanish concentrate, Chinese concentrate, and concentrate containing adulteration compounds). The lime juice samples were freeze‐dried and analyzed using FT‐IR spectroscopy. At first, principal component analysis (PCA) was applied for clustering, but the samples were not thoroughly clustered with respect to their original groups in score plots. To enhance the classification rates, different chemometric algorithms including variable importance in projection (VIP), partial least square‐discriminant analysis (PLS‐DA), and counter propagation artificial neural networks (CPANN) were used. The best discriminatory wavenumbers related to each class were selected using the VIP‐PLS‐DA algorithm. Then, the CPANN algorithm was used as a nonlinear mapping tool for classification of the samples based on their original groups. The lime juice samples were correctly designated to their original groups in CPANN maps and the overall accuracy of the model reached up to 0.96 and 0.87 for the training and validation procedures. This level of accuracy indicated the FT‐IR spectroscopy coupled with VIP‐PLS‐DA and CPANN methods can be used successfully for detection of authenticity of lime juice samples.

## Graphical abstract

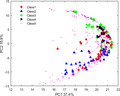

## 1 INTRODUCTION

There are many natural components in lime and lemon juice, such as Vitamin C, antioxidants, and anticancer compounds with great health benefits (Narang & Jiraungkoorskul, 2016; Patil et al., 2009). Lime juice is used in cooking and table‐top consumption. Lime is one of the major agricultural products in Iran, whose production in 2019 was about 633,000 tons (Anonymous, 2019). The considerable demand for lime juice and recent weather conditions has led to variability in the supply of fresh limes, giving unscrupulous producers the incentive to dilute their products with water and subsequent addition of citric acid to compensate for flavor loss.

According to Iranian National Standard, lime juice is obtained from fruit or is prepared with concentrate and water while manufacturers add the adulterant compounds into reconstructed lime juices. Nowadays, with increasing awareness of people, the consumption of beverages is not only for the taste, freshness, and thirst but also because of the health benefits and antioxidant compounds such as phenolic compounds and carotenoids (Zielinski et al., 2014). Foods and beverages have been intentionally or unintentionally contaminated with chemical compounds; however, added substances may not be harmful to human health, but are considered to be fraudulent (Everstine et al., 2013), and these compounds may cause allergic effects. The beverage industry has grown dramatically, and on the other hand, cheating in juices has also increased (Kelly & Downey, 2005). Fruit juice is one of the commonest industrial products that have been subjected to change and fraud (Abad‐García et al., 2014).

Fruit juice adulteration certainly has negative effects on juice quality. Moreover, with the global increase in adulteration, the impact of a single food adulteration event will affect a larger and wider population than ever. As contamination of lime juice with different compositions is an ongoing problem, appropriate analytical techniques are needed to detect frauds (Zhang et al., 2009).

Presently, there are high demands for the use of fast methods along with chemometrics for determination of fraud (Callao & Ruisánchez, 2018). The profiling methods such as Fourier transform infrared (FT‐IR), near infrared (NIR), and Raman spectroscopy would show the overall ingredients within a sample rather than looking for a single indicator component. FT‐IR spectroscopy has significantly developed to determine the biochemical patterns that can be processed by statistical classification methods such as principal components analysis (PCA), linear discriminate analysis (LDA), artificial neural networks (ANNs), and support vector machines (SVM) (Callao & Ruisánchez, 2018).

IR spectroscopy measures the vibrations of molecules and each functional group or structural feature of a molecule has a special vibrational frequency that indicates which functional groups are present within the samples. The characteristic spectra of samples provide a molecular “fingerprint” that can be used for classification purposes. Moreover, in this technique, relatively little sample preparation is needed, the whole analysis is rapid, cheap, and it can be easily employed in fundamental research and in the qualification control process. But, high‐performance liquid chromatography and gas chromatography have high costs (solvent consumption) and also require a long time for experimentation (Rodriguez‐Saona & Allendorf, 2011).

Multivariate data from FT‐IR need adequate statistical techniques to establish inferences among different samples concerning signal fluctuations. In this line, chemometric methods have been applied to evaluate the natural and geographical regions of foods, such as edible oils (Noorali et al., 2014), saffron (Petrakis et al., 2015), meat products (Pieszczek et al., 2018), wine (Fan et al., 2018), and juice (Ghaderi‐Ghahfarokhi et al., 2016).

There is a lot of information accumulated in FT‐IR spectra, and every single wavelength (number wave) contains special information about molecular structures. However, in many cases, a single wavelength cannot solve problems related to pattern recognition studies. Despite univariate approaches, multivariate methods are able to tackle recognition problems. One of the most important issues in multivariate analysis is the existence of a lot of independent variables usually obtained by different instruments. Variable selection can increase interpretability and accuracy of a model due to parsimonious representation (Farrés et al., 2015). One of the most efficient variable selection methods is the variable importance in projection–partial least squares (VIP‐PLS), which was introduced by Lennart et al. (2006). The PLS method is used to understand the correlation among all predictor variables (X) and response variables (Y), and the strength of the relationship between each predictor variable and the dependent variable is indicated by VIP index. The VIP values measure the importance of each variable used by a particular PLS model via their coefficients in every component (Rajalahti et al., 2009), and variables with VIP ≥ 1 are considered the most relevant to classify samples into different classes (Rajalahti et al., 2009).

ANN is among the most useful and applied chemometric methods, which can be used for clustering, classification, modeling of a property, noise filtering, predictions, modification of defects, and etc. (Moldes et al., 2017). ANNs have some advantages as follows: flexibility, compatibility, handling nonlinear relationships, and applicability to complex variables and predicaments (Astray et al., 2013). Counter propagation artificial neural network (CPANN) is one of the most commonly used versions of artificial neural networks that are applied for supervised classification purposes. Neurons of this network mimic the action of a biological neuron in response to an external stimulus (Ballabio et al., 2009).

The CPANN method is able to solve the nonlinear mapping problems which PCA and LDA are not able to solve (Melssen et al., 2006). The CPANN architecture has two layers of neurons: a Kohonen layer and an output layer (Grosberg layer) that performs the mapping of the multidimensional input data into the lower dimensional array (two dimensions) (Neiband et al., 2017). The Kohonen layer is composed of a grid of N^2^ neurons (where *N* is the number of neurons). The CPANN training process is similar to self‐organizing maps (SOM). The most similar neuron to the input vector is called the “winner neuron.” According to the location of the winner neuron in the Kohonen layer, the weights of the net are frequently updated on the basis of the input object. During training, all samples are placed in the mapping according to the assigned classes, and the number of the weights of the neurons is the same as the number of the variables in the original data. More detailed about the theory of CPANN algorithm can be found in literature (Neiband et al., 2017).

The purpose of this work was to determine lime juice fraud based on FT‐IR spectroscopy and different pattern recognition methods. Very diverse set of natural, synthetic and adulterated lime juice samples were prepared and used for development of the discriminative models. Since the number of wavenumbers in collected FT‐IR data was too much, the VIP‐PLS‐DA algorithm was used as a supervised dimension reduction technique. This method significantly filters the variables and removes redundant wavenumbers from original data matrix. The abstract space selected using VIP‐PLS‐DA algorithm was used as input for CPANN in order to development nonlinear classification models. In fact, the present contribution investigated for a list of functional groups and transmittance values which could make a reasonable discrimination between different classes of original and artificial lime juice samples.

## 2 MATERIALS AND METHODS

### 2.1 Chemicals

Ascorbic acid was purchased from Rankem, Co (Okhla). Citric acid, potassium bromide (Kbr), and sodium metabisulfite were purchased from Sigma Chemical Co. Brilliant Green was purchased from Hangzhou Mike Chemical Instrument Co. Ltd.

### 2.2 Sample reconstruction and collection

In this study, 101 lime juices were used for modeling, which can be divided into five general categories as follows: class 1, 9 samples of *Citrus limetta*; class 2, 8 samples of *Citrus aurantifolia,* both of which were cultivated in Shiraz; class 3, 8 samples prepared by Spanish concentrate (SC) from Rabei; class 4, 8 samples prepared by Chinese concentrate (CC) from Sunich; class 5, 68 synthetically adulterated samples that were prepared by lime juice concentrates (SC and CC), ascorbic acid (AA), citric acid (CA), metabisulfite (MBS), brilliant green, and lime waste (LW) (to increase phenolic compounds content) at different levels. Class 1 and class 2 samples are natural lime juice samples. The samples from class 3 and 4 are made based on Spanish and Chinese concentrates, and class 5 samples are artificially adulterated lime juice samples.

The unadulterated lime juices (samples of classes 3 and 4) were formulated based on the Iranian national standards of lime juice (INSO, 1996). Therefore, some of the indicators including color, pH, and Brix^o^ were adjusted by Hunter‐Lab scan XE–Spectrocolorimeter (Hunter Associates Laboratory), digital pH meter (Metrohm model 827), and refractometer DR‐D1 Atago at 20°C, respectively. The adulterated lime juices (samples of class 5) were formulated based on the most common frauds in Iran. The compositions of these samples (unadulterated and adulterated) are presented in Table 1.

**TABLE 1:** The compositions of different reconstructed lime juice samples in this work

Sample/ Composition		Water	LW mixed with water	CC (g)	SC (g)	CA (%)	AA (%)	MBS (ppm)	Salt/sugar (%)
Class 3	1	3 ml	—	—	0.48	—	—	—	—
	2	3 ml	—	—	0.51	—	—	—	—
	3	3 ml	—	—	0.49	—	—	—	—
	4	3 ml	—	—	0.52	—	—	—	—
	5	3 ml	—	—	0.46	—	—	—	—
	6	3 ml	—	—	0.51	—	—	—	—
	7	3 ml	—	—	0.50	—	—	—	—
	8	3 ml	—	—	0.47	—	—	—	—
Class 4	9	3 ml	—	0.31	—	—	—	—	—
	10	3 ml	—	0.30	—	—	—	—	—
	11	3 ml	—	0.29	—	—	—	—	—
	12	3 ml	—	0.28	—	—	—	—	—
	13	3 ml	—	0.27	—	—	—	—	—
	14	3 ml	—	0.26	—	—	—	—	—
	15	3 ml	—	0.25	—	—	—	—	—
	16	3 ml	—	0.24	—	—	—	—	—
Class 5	17	3 ml	—	—	0.41	2.4	—	—	—
	18	3 ml	—	—	0.37	2.4	—	—	—
	19	3 ml	—	—	0.39	2.4	—	—	—
	20	3 ml	—	—	0.39	2.4	—	600	—
	21	3 ml	—	—	0.42	2.4	—	—	—
	22	3 ml	—	—	0.41	1.2	1.2	—	—
	23	3 ml	—	—	0.37	1.2	1.2	—	—
	24	3 ml	—	—	0.39	1.2	1.2	—	—
	25	3 ml	—	—	0.42	1.2	1.2	—	—
	26	3 ml	—	—	0.45	1.2	—	—	—
	27	3 ml	—	—	0.46	1.2	—	—	—
	28	3 ml	—	—	0.43	1.2	—	—	—
	29	3 ml	—	—	0.44	1.2	—	—	—
	30	—	3 ml	—	0.40	—	—	—	—
	31	—	3 ml	—	0.36	—	—	—	—
	32	—	3 ml	—	—	6.5	—	—	—
	33	—	3 ml	—	—	7	—	—	—
	34	—	3 ml	—	—	7	—	600	—
	35	—	3 ml	—	—	6.5	1.2	300	—
	36	—	3 ml	—	0.36	1.2	1.2	300	—
	37	—	3 ml	—	0.33	1.2	1.2	600	—
	38	—	3 ml	—	0.37	1.2	1.2	300	—
	39	3 ml	—	—	0.39	1.2	1.2	600	—
	40	3 ml	—	—	0.40	1.2	1.2	300	—
	41	3 ml	—	—	0.46	—	—	—	0.5/0.5
	42	3 ml	—	—	0.48	—	—	—	0.5/0.5
	43	3 ml	—	—	0.42	1.2	1.2	—	0.5/0.5
	44	3 ml	—	—	0.33	2.4	—	—	0.5/0.5
	45	—	3 ml	—	—	6.5	1.2	300	0.5/0.5
	46	3 ml	—	—	0.24	6	—	600	—
	47	3 ml	—	0.24	—	2.4	—	—	—
	48	3 ml	—	0.25	—	2.4	—	—	—
	49	3 ml	—	0.23	—	2.4	—	—	—
	50	3 ml	—	0.27	—	2.4	—	—	—
	51	3 ml	—	0.26	—	1.2	1.2	—	—
	52	3 ml	—	0.27	—	1.2	1.2	—	—
	53	3 ml	—	0.24	—	1.2	1.2	—	—
	54	3 ml	—	0.25	—	1.2	1.2	—	—
	55	3 ml	—	0.28	—	1.2	—	—	—
	56	3 ml	—	0.27	—	1.2	—	—	—
	57	3 ml	—	0.26	—	1.2	—	—	—
	58	3 ml	—	0.24	—	1.2	—	—	—
Class 5	59	—	3 ml	0.19	—	2.4	—	—	—
	60	—	3 ml	0.21	—	2.4	—	—	—
	61	—	3 ml	0.18	—	2.4	—	—	—
	62	—	3 ml	0.19	—	1.2	1.2	—	—
	63	—	3 ml	0.18	—	1.2	1.2	600	—
	64	—	3 ml	0.21	—	1.2	1.2	300	—
	65	—	3 ml	0.22	—	1.2	1.2	300	—
	66	3 ml	—	0.24	—	1.2	1.2	300	—
	67	3 ml	—	0.26	—	1.2	1.2	300	—
	68	3 ml	—	0.25	—	1.2	1.2	600	—
	69	3 ml	—	0.22	—	1.2	1.2	—	0.5/0.5
	70	3 ml	—	0.25	—	1.2	1.2	—	0.5/0.5
	71	3 ml	—	0.24	—	2.4	—	—	0.5/0.5
	72	3 ml	—	0.22	—	2.4	—	—	0.5/0.5
	73	3 ml	—	0.18	0.24	—	—	—	—
	74	3 ml	—	0.15	0.28	—	—	—	—
	75	3 ml	—	0.18	0.24	1.2	1.2	300	—
	76	3 ml	—	0.15	0.28	1.2	1.2	—	—
	77	3 ml	—	0.18	0.24	2.4	—	—	—
	78	3 ml	—	0.15	0.28	—	—	—	—
	79	3 ml	—	0.18	0.24	2.4	—	—	0.5/0.5
	80	3 ml	—	0.15	0.09	5	—	300	—
	81	3 ml	—	0.18	0.09	5	—	—	—
	82	—	3ml	0.13	0.24	1.2	1.2	—	—
	83	—	3ml	0.15	0.21	2.4	—	600	—
	84	—	3ml	0.13	0.24	2.4	—	—	—

Note

Class 1: *Citrus limetta*; Class 2: *Citrus aurantifolia*; Class 3: Spanish concentrate; Class 4: Chinese concentrate; Class 5: Concentrates with additional composition.

Abbreviations: AA, Ascorbic acid; CA, Citric acid; CC, Chinese concentrate; CS, Spanish concentrate; LW, lime waste which is mixed with water; MBS, Metabisulfite.

Finally, after development of CPANN classification models, 20 brands of lime juices (collected from local markets) were evaluated and predicted based on the presented model.

### 2.3 Preparation of samples for FT‐IR spectroscopy

Two ml of each sample was dried with Christ Alpha 1–2 LD Plus (Germany) freeze dryer at −55°C and pressure of 1 mbar for 36 hr. Then, 1 mg of the dried lime juice was blended with potassium bromide powder (KBr) and compressed as tablets. The FT‐IR spectra were obtained with Nicolet IR 100 (Thermo Scientific). All spectra were rationed against a background air spectrum and gathered as transmit values at each data point. Each sample was replicated 5 times, and the spectral resolution was set at 8 cm^‐1^.

### 2.4 Multivariate statistical analysis

The spectral information was saved as.xlx files by Encompass program to be analyzed with MATLAB software, version 8.3.0.532. The IR transmittances were recorded in 468 wavenumbers, and the size of the collected data matrix was 505 × 468. The collected FT‐IR data were mean centered and then autoscaled according to the standard deviation of the wavenumbers. For this standardization, the FT‐IR information (D_1_) was transformed into the D_2_ value according to the following equation (Neiband et al., 2017):

$$D_{2}=\frac{D_{1}‐D_{m}}{\delta_{s}}$$

Where *D_m_* and *δ_s_* are the mean and the standard deviation of the FT‐IR spectra for each wavenumber, respectively.

In this study, at first, the PCA method was applied for clustering of the lime juice samples. The results of PCA analysis will give a preliminary view on the collected data and will guide for deciding about the selection of the appropriate classification algorithm for discrimination of samples.

The best discriminatory variables in this work were selected using VIP‐PLS‐DA algorithm, and subsequently, the samples were classified according to variables with the VIP > 1. These variables were used as inputs for development of CPANN models. The number of neurons (NN) and training epochs (NE) were optimized using the classification toolbox in MATLAB. The optimized NN and NE were equal to 20 and 200, respectively. Eventually, the performance of the CPANN model was compared to those of PLS‐DA, and the models were evaluated using an external test set and Y‐randomization test.

## 3 RESULLTS

### 3.1 Spectral exploration

**FIGURE 1:**
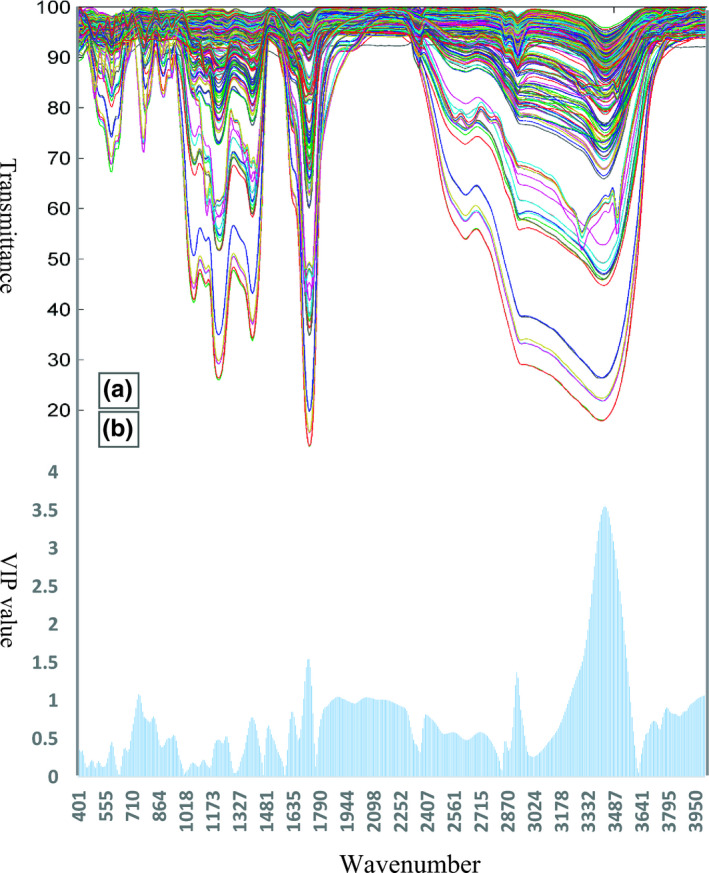
(a) The FT‐IR spectra for different lime juice samples in transmittance mode, (b) The values of VIP index calculated for each wavenumber using VIP‐PLS‐DA algorithm

The FT‐IR spectra of different samples are shown in Figure 1a. As can be seen in this figure, all spectra are rather complex and multivariate statistical methods are needed to explore the differences between the samples.

**FIGURE 2:**
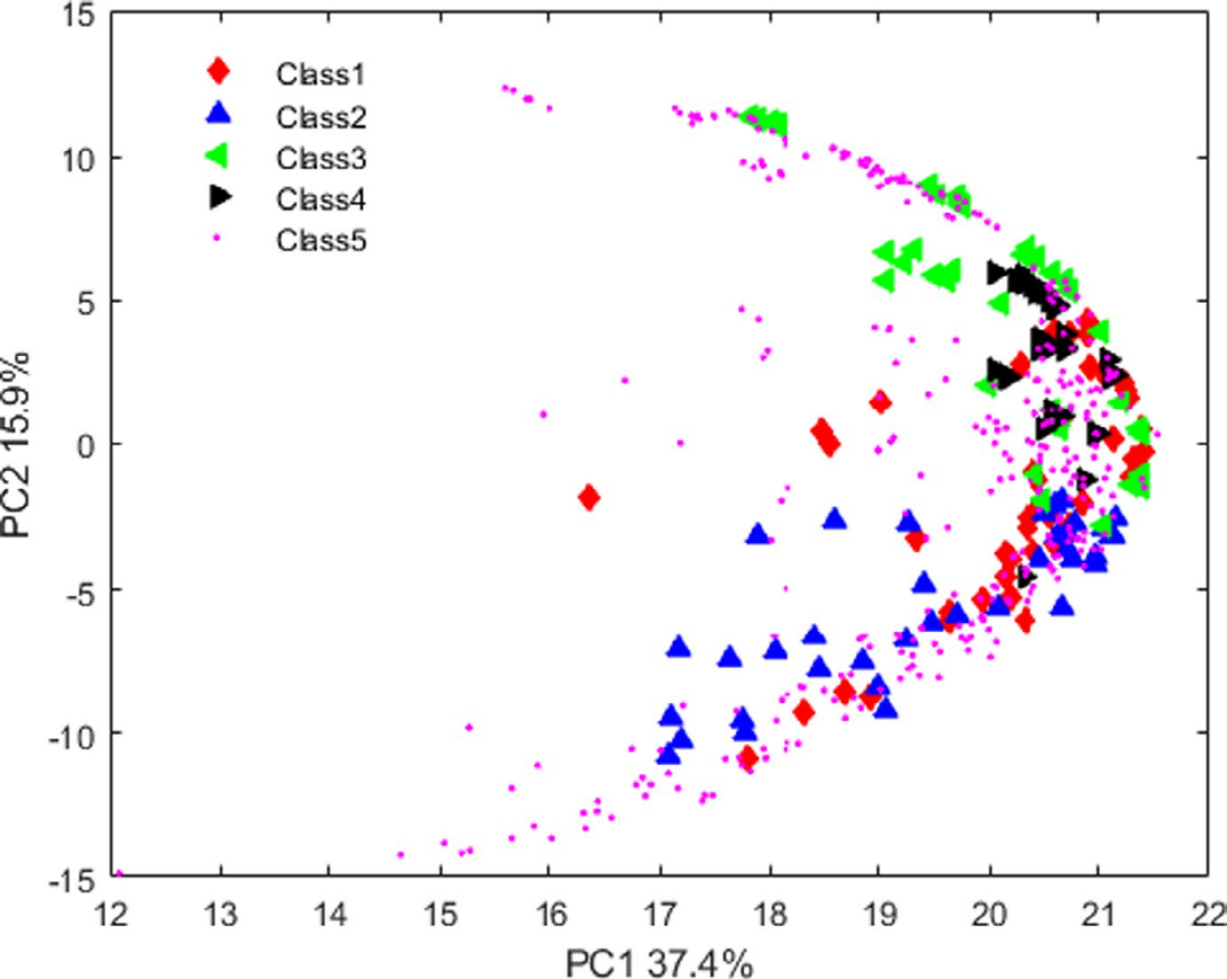
Representation of the values of PC1 against PC2 for different lime juice samples

The intense bond in the region of 2500–3500 cm^‐1^ shows the existence of free and intermolecular bonded hydroxyl groups of carboxylic acids. The functional groups of –COOH and –COOCH_3_ are indicated at 1727 cm^‐1^. The peak of 1,257 cm^‐1^ is attributed to the CO groups of aliphatic acids. The bands of the fundamental molecular vibrations of CC, CO, and CCO of sugar are assigned at 1,050 cm^‐1^. The rocking CH_3_ of pectin was observed at 950 cm^‐1^. The bond related to the region of 500–600 cm^‐1^ is attributed to the aromatic compounds (Andronie et al., 2017). As a preliminary multivariate analysis, the PCA method was used to evaluate the spectroscopic data. The score plot of the first and second PCs is illustrated in Figure 2. The first two PCs explain 53.3% of the variance of the dataset. As can be seen in Figure 2, samples of class 2 are seasonably separated from samples of classes 3 and 4. On the other hand, samples of class 1 are not well separated and this group has heavy overlapping with samples of classes 2, 3, and 4. Moreover, the samples of class 5 have extreme overlapping with all groups of samples in PCA score plot. The data shown in score plot revealed that although some groups of samples are separated in PC space but there are still high degrees of overlapping between them in this space.

Since the PCA algorithm was not able to separate samples thoroughly, more robust pattern recognition algorithms were used for dimension reduction and mapping of the multivariate dataset. In this investigation, initially, the important variables were selected and extracted using the VIP‐PLS‐DA method. The calculated VIP values associated with each wavenumber are shown as bar plots in Figure 1b. The effective wavenumbers which had a VIP value greater than 1 were considered for classification (102 variables). This reduced space is information rich for discriminating between different groups of lime juice samples. Detailed view of Figure 1b reveals that the spectral region of 3,200 to 3,600 cm^‐1^ had a significant influence for discrimination among groups of samples. Moreover, six other regions including 741–756, 1697–1743, 1859–1944, 2013–2198, 2916–2939, and 3950–4003 cm^‐1^ revealed high VIP values and showed a great effect in classification of different groups.

### 3.2 Counter propagation artificial neural networks coupled with VIP‐PLS‐DA analysis

**FIGURE 3:**
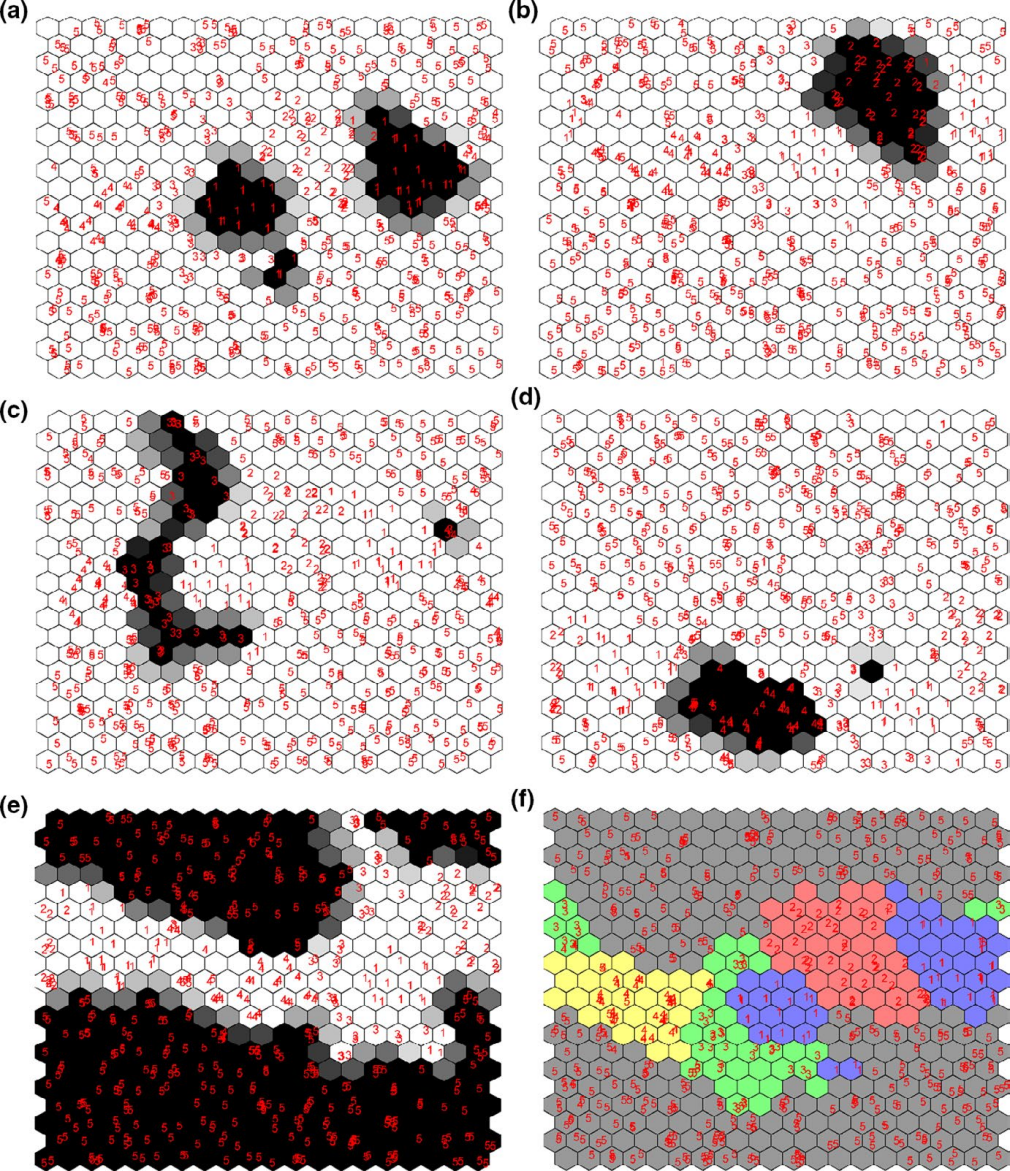
The top map of the trained CPANN model for classification of lime juices weighted for (a) *Citrus limetta*, (b) *Citrus aurantifolia*, (c) Spanish concentrate, (d) Chinese concentrate, (e) concentrate containing adulterant compounds, and (f) CPANN map for each class that has its own color

CPANN is a nonlinear process that classifies samples according to the similarity of their independent variables. This method can detect the relationship between input and output data and uses it to classify and annotate samples onto the final map. In order to develop the CPANN models, the VIP‐selected wavenumbers were used as inputs and NN and NE were set to 20 and 200, respectively. The color‐coded top maps of the developed CPANN model in this work are illustrated in Figure 3. Each map is weighted according to the groups of the collected lime juices. As can be seen in this figure, the groups of lime juices are well separated and similar samples are annotated to neighbor clusters of neurons. After the model development procedure, the accuracy, specificity, sensitivity, and prediction power of the models were investigated (Ballabio et al., 2018; Gondim et al., 2017; Lopez et al., 2015). The results of CPANN models in this work for the training and validation procedure are summarized in Table 2. As can be seen in Table 2, the accuracy of the model is reasonable (96%), and the other classification parameters (i.e., specificity, sensitivity, and precision) revealed high power of the model for detection of frauds in lime juice samples. It is worth to mention that the sensitivity and precision rates for class 4 are lower than those of other classes, which was probably due to the presence of additives such as metabisulfite, ascorbic, and citric acids in the Chinese concentrate.

**TABLE 2:** The statistical parameters of the CPANN model developed for classification of lime juice samples

Samples	Train set		Venetian Blind Cross‐Validation (*n* = 5)	
	Specificity/sensitivity/ precision	Overall accuracy/NER/ER	Specificity/sensitivity/ precision	Overall accuracy/NER/ER
Class 1	1.00/0.93/0.98	0.96/0.94/0.06	0.97/0.69/0.70	0.87/0.80/0.20
Class 2	1.00/1.00/0.98		0.99/0.85/0.85	
Class 3	0.99/1.00/0.87		0.98/0.88/0.78	
Class 4	0.99/0.80/0.84		0.97/0.68/0.69	
Class 5	0.96/0.97/0.98		0.85/0.92/0.93	

Note

Class 1: *Citrus limetta*; Class 2: *Citrus aurantifolia*; Class 3: Spanish concentrate; Class 4: Chinese concentrate; Class 5: Concentrates with additional composition.

Abbreviations: ER, error rate; NER, nonerror rate.

The confusion matrices of the CPANN model for training and validation procedures are given in Table 3. As can be seen in Table 3, while almost all the samples were classified in their own groups, some samples of class 4 were categorized in class 5, which is due to the similarity between these classes. Furthermore, some samples of class 1 were classified in class 5, which is probably due to the specific composition of some samples in class 5, which contained lime waste and concentrates.

**TABLE 3:** Confusing matrices of CPANN model for the training and validation procedures

Class No.	Training					Venetian blind cross‐validation (*n* = 5)				
	Class 1	Class 2	Class 3	Class 4	Class 5	Class 1	Class 2	Class 3	Class 4	Class 5
Class 1	42	1	0	0	2	31	1	2	0	11
Class 2	0	40	0	0	0	3	34	0	0	3
Class 3	0	0	40	0	0	3	0	35	1	1
Class 4	0	0	3	32	5	0	0	3	27	10
Class 5	1	0	3	6	330	7	5	5	11	312

Note

Class 1: *Citrus limetta*; Class 2: *Citrus aurantifolia*; Class 3: Spanish concentrate; Class 4: Chinese concentrate; Class 5: Concentrates with additional composition.

**FIGURE 4:**
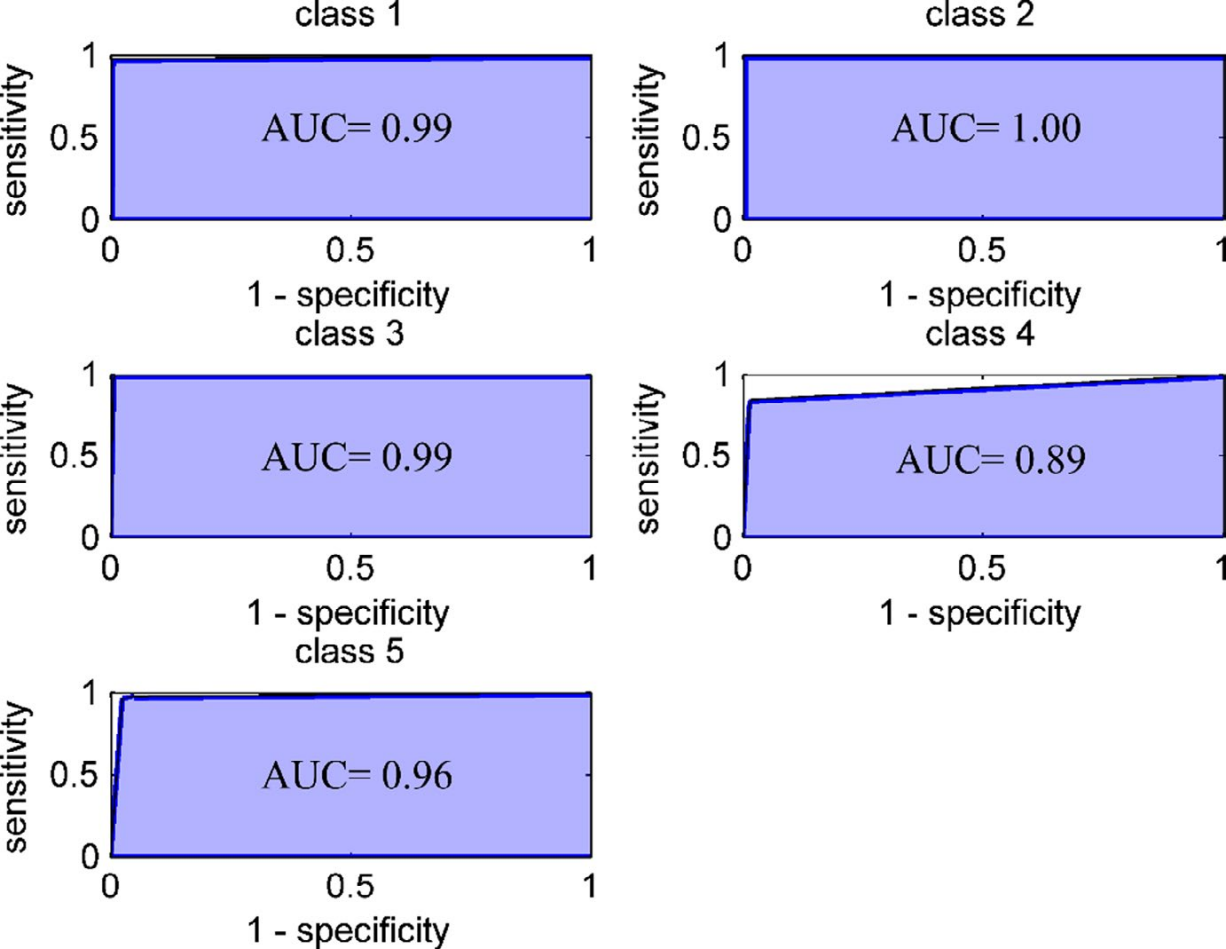
Receiver operating characteristic curves for discrimination of five groups of lime juice samples. The AUC values are shown as legends in the figures

The prediction ability of the developed CPANN model was more evaluated using the receiver operating characteristic curves (ROC). The area under the ROC curve (AUC) can be used as a measure for addressing the discriminatory power of the classification models. The ROC curves of CPANN model annotated to each groups of samples are illustrated in Figure 4. The prediction ability was acceptable for all classes although for class 4, it was lower than the others. This is in agreement with those seen in Tables 2 and 3 for this class of samples.

### 3.3 Comparison with conventional linear classifier and external validation

In order to compare the CPANN model with other methods, the PLS‐DA analysis was also conducted in this research for classification of samples. After selection of discriminatory variables using VIP approach, the dataset including the selected variables was used as an input for conventional linear PLS‐DA method. The results of VIP‐PLS‐DA model for classification of samples were compared with those obtained for the CPANN model. The results of VIP‐PLS‐DA approach are given in Table 4. The parameters related to training of the PLS‐DA model including accuracy, nonerror rate, and error rate were equal to 0.75, 0.46, and 0.54, respectively, and these values for the validation procedure were 0.75, 0.44, and 0.56. These results indicate that the performance of the CPANN model is superior over linear PLS‐DA approach. This observation is due to the potential nonlinear relationships in collected dataset.

**TABLE 4:** The statistical parameters of the PLS‐DA model developed for classification of lime juice samples

Samples	Train set		Test set	
	Specificity/sensitivity/ precision	Overall accuracy/NER/ER	Specificity/sensitivity/ precision	Overall accuracy/NER/ER
Class 1	0.99/0.20/0.75	0.75/0.46/0.54	1.00/0.16/0.78	0.75/0.44/0.56
Class 2	0.99/0.25/0.67		0.98/0.20/0.53	
Class 3	1.00/0.00/0.00		1.00/0.00/0.00	
Class 4	0.98/0.88/0.78		0.98/0.88/0.78	
Class 5	0.36/0.96/0.76		0.35/0.96/0.75	

Note

Class 1: *Citrus limetta*; Class 2: *Citrus aurantifolia*; Class 3: Spanish concentrate; Class 4: Chinese concentrate; Class 5: Concentrates with additional composition.

ER, error rate; NER, nonerror rate.

For a better illustration of the functionality of the built CPANN model, Y‐randomization test was used. This validation method examines the possibility of the chance correlation and evaluates the reliability and robustness of the model by permutation testing. The dependent variable (Y) was randomized, and a new model was fitted using the dataset. As a result, the obtained model should be of poor quality and without real meaning (randomization). In this research, 10 random shuffles of the Y vector were performed and the average classification measures were reported. The detailed results of the Y‐randomization test are given in Table 5. The statistical values given in this Table indicate that the developed CPANN model in section 3.2 is robust and good classification accuracies are not by chance. This conclusion is valid, since all performance measures of the randomized model were lower compared with those of the nonrandomized model.

**TABLE 5:** The statistical parameters of the randomized CPANN model for classification of samples

Samples	Train set		Test set	
	Specificity/sensitivity/ precision	Overall accuracy/NER/ER	Specificity/sensitivity/ precision	Overall accuracy/NER/ER
Class 1	0.98/0.56/0.71	0.80/0.61/0.38	0.90/0.07/0.06	0.49/0.20/0.80
Class 2	0.98/0.47/0.70		0.91/0.03/0.02	
Class 3	1.00/0.38/0.88		0.94/0.10/0.13	
Class 4	0.95/0.75/0.57		0.91/0.17/0.14	
Class 5	0.67/0.94/0.86		0.36/0.67/0.68	

Note

Class 1: *Citrus limetta*; Class 2: *Citrus aurantifolia*; Class 3: Spanish concentrate; Class 4: Chinese concentrate; Class 5: Concentrates with additional composition.

ER, error rate; NER, nonerror rate.

## 4 DISCUTION

This research showed that FT‐IR data coupled with the VIP and CPANN model could be an effective method for frauds detection, while researchers were looking for detection of specific compound or ratio between compounds as an indicator for fraud detection. European Fruit Juice Association measured some of the indicators, such as Brix, relative density, citric acid, D‐isocitric acid, ash, flavonoids (hesperidin and eriocitrin), glucose, and fructose, for detection of fraud in lime juice. However, these indicators are not sufficient to determine fraud (AIJN, 2015). Lorente et al., (2014) reported that these indicators were not appropriate for fraud detection in Spanish lemon juice because some of the parameters were not reached or exceeded from the range determined by European Fruit Juice Association. However, they confirmed that some of the measured parameters such as D‐isocitric acid, citric acid, and potassium ion concentration could differentiate between natural and reconstructed groups of lime juices.

The list of some analytical methods for determination of fraud in lime juice samples is given in Table 6. In this regard, Asemi et al., (2008) measured phenolic compounds and flavonoids by optical rotation and spectroscopy to identify adulteration of lime juices. Alizadeh et al. (2017) also used GC to identify fraud by determination of volatile organic compounds. In another study, Guyon et al., (2014) studied the simultaneous determination of carbon 13 isotope ratio (δ^13^C/^12^C) of citric acids, glucose, and fructose in lime juices. The results of their study showed that lemon juice fraud can be determined using these ratios with a confidence level of 95%. However, Haminiuk et al., (2012) and Wang and Jablonski (2016) indicated that phenolic and flavonoids compounds could not be a good indicator for fraud detection because growth conditions, rainfall, dewatering methods, and other factors can affect the amount of these compounds. Therefore, in addition to being expensive and difficult, these methods may not be able to detect fraud in very diverse sets of samples.

**TABLE 6:** Some of the appropriate methods for determination of frauds in lime juice samples

Number of samples	Kind of samples	Method	Indicator	Multi/univariate	Results	References
___	___	GC	Volatile organic compounds	Univariate (The polynomial models)	Good method for fraud detection (R^2^ = 95%)	Alizadeh et al. (2017)
65	Natural (35) and commercial (30)		Carbon isotopic, glucose and fructose	Univariate	Determine the range for each indicator	Guyon et al. (2014)
25	squeezed lemon juice	LC‐MS	LC‐MS datasets (Eriocitrin, Hesperidin, Diosmin, Genistein, Gentiopicroside)	Multivariate (PCA)		Wang and Jablonski (2016)
39	Natural (1) and commercial (38)	NIR	Functional groups	Multivariate (SVM, PCA)	The superiority of SVM to PCA for classification	Shafiee and Minaei (2018)
74	Natural (74)	ICP‐MS	25 trace elemental (Ag, Al, As, Ba, Bi, Cr, Cu, Fe, Ga, etc.)	Multivariate (LDA, k‐NN, PLS‐DA, SVM and RF)	SVM had better performance compared to other methods	Gaiad et al. (2016)
101	Natural (17), synthetic (16), synthetic‐containing adulteration (68)	FT‐IR	Functional groups	Multivariate (VIP‐CPANN)	High accuracy of model (96%) for classification	This work

Another effective way to identify fraud and authenticity is to use inexpensive techniques (FT‐IR and NIR) in conjunction with chemometrics, which are considered as effective methods for investigating a list of functional groups. In this regard, Shafiee and Minaei (2018), using NIR and chemometrics, investigated the originality of lime juices. The results of their work showed that PCA as an unsupervised method is not always suitable, while supervised methods such as SVM after variable selection were effectively able to classify and distinguish classes (more details are given in Table 6). However, this study is not comprehensive enough to determine authenticity due to the limited number of the samples.

Another method used to detect fraud was inductively coupled plasma‐mass spectrometry (ICP‐MS) for determination of twenty‐five elements. The results indicated that this method was able to attribute unknown lemon juice samples to their geographical origins. SVM had a better performance in this type of classification compared to RF (random forests), k‐NN (k‐Nearest Neighbor), LDA, and PLS‐DA (Gaiad et al., 2016).

In addition, the use of spectroscopic techniques coupled with chemometrics plays a major role to detect authenticity of juices. Hohmann et al., (2014) applied ^1^H‐NMR to detect organically produced tomatoes. Tomato samples of two different cultivars from four different producers over a seven‐month period were analyzed. LDA was performed and revealed significant differences among the growing regions, and external validation confirmed 100% accuracy for the classification of tomato samples. Hirri et al., (2016) used PCA and PLS‐DA to FT‐IR spectral data to detect geographical origins of olive oil from Morocco (Beni Mellal, Agadir, and Berkan). They performed PCA and PLS‐DA on the FT‐IR spectral data from the juice samples and reached 100% accuracy in classification of the three geographical origins.

The FT‐IR spectroscopy as a screening technique, in contrast with HPLC and HPLC‐CO‐MS, is inexpensive, fast, easy, adaptable with chemometrics and can provide information on functional groups to classify samples. In this study, the collected FT‐IR spectra were mathematically explored by the VIP method. In this process, the information‐rich wavenumbers of FT‐IR spectra that effectively created differences between the groups were selected. Based on the calculated VIP values, the important variables were separated from the whole data and a CPANN model was built using these wavenumbers as inputs.

In the CPANN model, according to the existing differences among the groups, the samples were categorized in five classes. The results of the sensitivity and specificity of each class were reasonable and highly reliable. In fact, sensitivity is the ability of the model to detect its samples, while specificity is the ability of the model to distinguish external samples. A closer look at the results reveals that the developed CPANN model was able to recognize true positive and true negative samples for all classes, simultaneously.

The statistical performance of the CPANN model was compared with PLS‐DA and the results showed that many samples were misclassified, using PLS‐DA approach. The PLS‐DA model was not able to correctly fit lime juice samples. Unlike PLS‐DA, the CPANN results showed good discrimination for all classes. The accuracy, nonerror rate, and error rate reached up to 96%, 94%, and 6% for the training procedure, respectively. The nonerror rate for validation procedure was 87% which is valuable in fraud detection in lime juice industry. Superiority of CPANN model over PLS‐DA confirmed the existence of a nonlinear relationship between the dependent and independent variables, in this work. It meant that there was a complex relationship between the variables (functional groups) for classification, which was not clearly understood using PLS‐DA. However, CPANN correctly modeled these complex relationships.

In order to demonstrate that good results of CPANN model were not by chance, Y‐randomization was performed. The results of this test confirmed the robustness of the developed model in this work (Kiralj & Ferreira, 2009; Tropsha & Golbraikh, 2007).

In addition, another advantage of this study was the variety and high diversity of the samples. With very diverse sets of samples, the classification model will be more accurate and reliable. In this research, the CPANN model was trained with diverse set of samples and was able to predict the lime juices collected from different origins. Although most studies have focused on fruit juice dilution (Dasenaki & Thomaidis, 2019), geographical origin (Gaiad et al., 2016), mixing with other inexpensive fruit nectars (Naderi‐Boldaji et al., 2018), and mixing original concentrates with glucose syrup (Yaman & Durakli Velioglu, 2019), this study, for the first time, sought to investigate some of the most common frauds in Iran by identifying unauthorized additives (such as lime waste, citric acid, ascorbic acid, metabisulfite, salt, and sugar) using an inexpensive and precise method. Therefore, this work was not limited to natural lime juices and synthetic samples, as it also dealt with a number of dangerous frauds (more details are given in Table 6).

Finally, in order to check the market samples, 20 brands were evaluated based on their FT‐IR spectra and the trained CPANN model. Interestingly, all samples were thoroughly categorized in class 5 (accuracy = 100% for validation set), which could be a warning to the food industry and consumers. The presence of unauthorized components (metabisulfite) in lime juice will cause harm to consumers (such as allergic reactions and dyspnea) and will also reduce the credibility of the food industry.

## 5 CONCLUSIONS

In this work, the authenticity of commercial lime juice was detected and quantified using FT‐IR spectroscopy coupled with the VIP variable selection and CPANN models. The main advantage of the present contribution is the diversity of the calibrating samples which include broad ranges of natural, synthetic, and adulterated lime juice samples. Therefore, applicability domain of the developed discriminative model in this work would be broad and wide which is a needed property in fraud detection in lime juice industry. The calculated VIP values indicated that the molecular functional groups with IR signals in the ranges of 741–756, 1697–1743, 1859–1944, 2013–2198, 2916–2939, and 3950–4003 cm^‐1^ are important to detect fraud in lime juice samples. The classification results showed that FT‐IR spectroscopy in conjunction with CPANN model has a great potential to be used as an alternative method for screening purposes and detection of frauds in lime juice industry. Moreover, the developed model was validated using Venetian blind cross‐validation and using Y‐randomization tests. The CPANN model was also compared with conventional PLS‐DA algorithm. The results indicated that the construction of the model was successful, and good classification results were not due to chance correlation. Therefore, the developed CPANN model in this work can be of interest for food quality control institutions and food safety organizations to reduce lime juice frauds.

## ETHICAL APPROVAL

On behalf of all coauthors, I, Dr. Mohsen Barzegar, declare that this article has not been published in or is not under consideration for publication elsewhere. All authors were actively involved in the work leading to the manuscript and will hold themselves jointly and individually responsible for its content. Also, there is no conflict of interest in this paper.

## Data Availability

The data that support the findings of this study are available from the corresponding author upon reasonable request.
